# Study protocol for the Bio-HEAT study: Investigating the Biological pathways from HEAT exposure to preterm birth and other adverse maternal and child health outcomes in South Africa

**DOI:** 10.12688/wellcomeopenres.23616.1

**Published:** 2025-03-05

**Authors:** Ijeoma Solarin, Darshnika Pemi Lakhoo, Kimberly Mc Alpine, Margaret M. Brennan, Admire Chikandiwa, Nicholas B. Brink, Lebohang Radebe, Marié Landsberg, Clive Gray, G Justus Hofmeyr, Howard Chang, Robyn Hetem, Sibusisiwe Makhanya, Phelelani T. Mpangase, Shane Norris, Michael Urban, Valerie Vannevel, Amy Wise, Matthew F. Chersich, Karl-Gunter Technau, Renate Strehlau

**Affiliations:** 1 Wits PHR, University of the Witwatersrand Johannesburg Faculty of Health Sciences, Johannesburg, Gauteng, South Africa; 2 Department of Public Health and Primary Care, Institute of Population Health, Trinity College Dublin School of Medicine, Dublin, Leinster, Ireland; 3 Reproductive Immunology Research Consortium in Africa (RIRCA), Division of Molecular Biology and Human Genetics, Faculty of Health Sciences, Stellenbosch University, Stellenbosch, Western Cape, South Africa; 4 Effective Care Research Unit, University of the Witwatersrand Johannesburg School of Clinical Medicine, Johannesburg, Gauteng, 2000, South Africa; 5 Department of Obstetrics and Gynaecology, University of Botswana, Gaborone, South-East District, Botswana; 6 Department of Biostatistics and Bioinformatics, Emory University Rollins School of Public Health, Atlanta, Georgia, 30329, USA; 7 Faculty of Science, University of Canterbury School of Biological Sciences, Christchurch, Canterbury, New Zealand; 8 IBM Research Africa, Johannesburg, Gauteng, South Africa; 9 Sydney Brenner Institute for Molecular Bioscience, University of the Witwatersrand Johannesburg Faculty of Health Sciences, Johannesburg, Gauteng, South Africa; 10 Wits Developmental Pathways for Health Research Unit (DPHRU), Department of Paediatrics and Child Health, School of Clinical Medicine, University of the Witwatersrand Johannesburg Faculty of Health Sciences, Johannesburg, Gauteng, South Africa; 11 Division of Human Genetics, National Health Laboratory Service and School of Pathology, University of the Witwatersrand Johannesburg Faculty of Health Sciences, Johannesburg, Gauteng, South Africa; 12 Department of Obstetrics and Gynaecology, Tygerberg Hospital, Stellenbosch University Faculty of Medicine and Health Sciences, Cape Town, Western Cape, South Africa; 13 Dept of Obstetrics and Gynaecology, Rahima Moosa Mother and Child Hospital, University of the Witwatersrand Johannesburg School of Clinical Medicine, Johannesburg, Gauteng, South Africa; 14 Empilweni Services and Research Unit, Rahima Moosa Mother and Child Hospital, Department of Paediatrics and Child Health, University of the Witwatersrand Johannesburg Faculty of Health Sciences, Johannesburg, Gauteng, 2093, South Africa; 15 WITS VIDA, Nkanyezi Research Unit, Department of Paediatrics, Rahima Moosa Mother and Child Hospital, Department of Paediatrics and Child Health, University of the Witwatersrand Johannesburg Faculty of Health Sciences, Johannesburg, Gauteng, 2093, South Africa

**Keywords:** Hot Temperature, Inflammation, Pregnancy, Premature birth, Maternal health, Breastfeeding, Infant health, Epigenetics, Biological markers, South Africa

## Abstract

**Introduction:**

Epidemiological evidence linking heat exposure to adverse maternal and child health outcomes is compelling. However, the biological and social mechanisms underlying these associations remain poorly understood. Understanding the pathways explaining these associations is important given rising global temperatures, and the urgent need for developing and testing adaptive interventions.

**Methods:**

This transdisciplinary study in Johannesburg, South Africa, will monitor a cohort of 200 women from their second trimester until one-year postpartum, alongside their infants. Heat exposure and environmental factors will be tracked using personal, community and facility-level temperature monitors and geospatial data. Data will be collected on social conditions, medical and obstetric history, heat stress and adaptation, hydration, mental wellbeing, and sleep quality. Clinical data includes physical measurements, ultrasound, cardiotocography, and biological specimens (blood, urine, saliva) analysed for inflammatory markers, RNA, metabolic indicators, renal function and hormonal levels. Placental and cord blood analyses will assess foetal stress. Infant data will include medical history, hospital visits, neurodevelopment, anthropometric measurements, vital signs, and urine analysis. Three nested sub-studies (20–50 participants) will explore specific aspects: Sub-study 1 will use wearable devices to monitor sleep, activity, and heart rate in high-risk women; Sub-study 2 will involve qualitative interviews; and Sub-study 3 will assess breastmilk composition and volume.

**Planned analyses:**

Our primary aim is to document linkages between heat exposure and inflammatory pathways that precede preterm birth. The hypothesis that heat exposure triggers maternal inflammation will be tested by analysing epigenetic changes associated with inflammatory cytokine protein and gene expression. We will investigate thermoregulation and hydration during labour. Using isotope techniques, we assess whether heat exposure alters breastmilk composition and volume. Conceptual frameworks and graphical causal models will be developed to delineate pathways of vulnerability and protective mechanisms.

## PLAIN LANGUAGE SUMMARY

Climate change is causing global temperatures to rise, leading to more frequent and intense heatwaves. This extreme heat can have health risks for pregnant women and their babies such as preterm birth and low birth weight. However, we do not yet fully understand how pregnant women’s body’s respond to heat, or why these health risks occur. Gaining this knowledge will help us to protect mothers and babies as the world gets warmer.

The Bio-HEAT study will follow 200 pregnant women from an underserved urban community in Johannesburg, South Africa. Heat exposure in the area is worsened by the Urban Heat Island effect where buildings, road and industry trap heat. Many people in this community don’t have access to cooling systems like air conditioning, and some use solid fuels like wood and coal for cooking, which makes their homes hotter. These conditions make them especially vulnerable to heat.

Our main goal is to understand how heat exposure might trigger inflammation and other changes in the body and lead to preterm birth. Women will be followed from their second trimester through to one year postpartum, along with their infants. Researchers will collect information through questionnaires, biological samples (like blood, urine and saliva), ultrasound scans, and wearable devices that measure heat exposure. Placenta and cord blood will be assessed for the effects of heat on babies before birth. The babies growth and health will also be monitored.

The study includes three smaller sub-studies: one to monitor activity, sleep and heart rate; another to gather personal experiences, and a third to explore how heat affects breastfeeding.

The findings from the study will help to inform strategies to reduce heat-related health risks for pregnant women and babies in similar urban settings globally.

## Introduction

Climate change, driven by anthropogenic greenhouse gas emissions, is one of the greatest threats to global health ^1^. Rising global temperatures are one of the most concerning manifestations of climate change, resulting in higher seasonal temperatures and more frequent, intense heatwaves ^2^. According to NASA, 2024 was the warmest year on record, approximately 1.47°C above the pre-industrial average ^3^. Global temperatures are projected to rise substantially further by 2070, potentially exposing one-third of the global population to mean annual temperatures exceeding 29°C – conditions currently limited to 0.8% of the Earth’s land surface ^4^.

Recently, pregnant women, foetuses and neonates have been identified as particularly vulnerable to the increasing ambient temperatures ^5^. Humans regulate their core temperature within a narrow range of 35.5–37.5°C ^6^, by balancing heat loss through behavioural and physiological mechanisms, with heat gain from metabolic (internal) and environmental (external) sources ^7^. Physiological and anatomical changes during pregnancy may impair normal thermoregulation, increasing susceptibility to heat-related stress ^5^. Heat exposure during pregnancy has been linked to several adverse pregnancy outcomes including hypertensive disorders of pregnancy ^8, 9^, pre-term birth ^8, 10^, low birthweight ^8, 10^, reduced foetal growth ^8, 11^, stillbirths ^8, 10^, and congenital anomalies ^8, 12^. Heat exposure in utero has also been associated with a range of detrimental health and social outcomes across the life-course ^13^. Underlying biological mechanisms have not yet been characterised, but are hypothesised to include elevated maternal core temperatures ^14^, endocrine dysfunction ^14^, inflammation ^14^, epigenetic changes ^7^, dehydration ^7, 14^, disrupted breastfeeding ^15, 16^, and compromised placental development ^7, 10, 14^. High ambient temperatures and adverse pregnancy outcomes are of particular concern in climate change hotspots, such as sub-Saharan Africa ^1, 17^. Despite some progress in recent decades, Africa ranks among the regions with the highest rates of maternal mortality, preterm births and other adverse pregnancy outcomes worldwide ^18, 19^, carrying significant implications for health and development.

Africa is disproportionately vulnerable to the effects of climate change, owing to its warm and dry climate with variable rainfall patterns, and low adaptive capacity ^20^. Southern Africa, in particular, is a climate-change hotspot, with temperatures rising at about twice the global rate and more frequent heatwaves of unprecedented intensity ^2, 21^. In a future with low mitigation efforts, the region is projected to become significantly hotter and drier, with even more variable rainfall ^22– 24^. Furthermore, sub-Saharan Africa remains a region with persistently high birth rates ^25^, and projections indicate that the paediatric population will be the largest in the world by 2055, surpassing 1 billion. Rapidly rising temperatures and other climate-related hazards threaten to exacerbate these challenges, further straining maternal and child health programs ^26– 28^.

Current global efforts to reduce emissions fall significantly short of the scale and pace required to avert dangerous climate change ^29^. Between 2030 and 2050, climate change is expected to cause approximately 250 000 additional deaths per year from malnutrition, malaria, diarrhoea and heat stress ^30^. Implementing adaptation measures alongside mitigation efforts is therefore crucial to reduce climate change-related mortality and morbidity. However, increased understanding of the biological effects of heat in humans is essential for designing effective, targeted adaptive interventions to protect vulnerable populations such as pregnant women, foetuses and neonates ^8, 14^.

### Research aims

The Bio-HEAT study aims to explore the underlying associations between heat exposure and the biological and social pathways that contribute to preterm birth, influence intrapartum outcomes, and impact breastfeeding.

### Hypotheses

**Figure 1.**
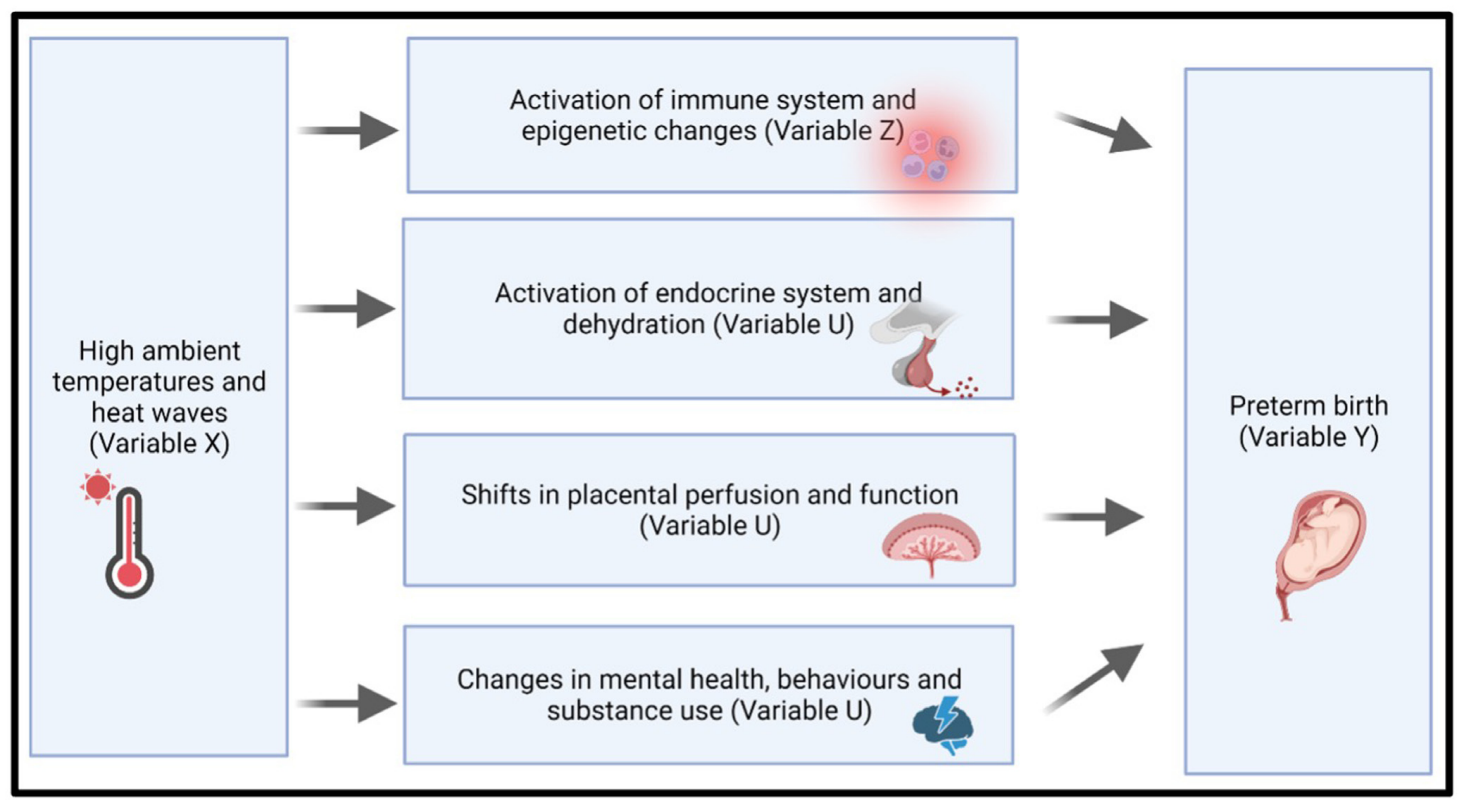
Proposed biological pathways linking heat exposure to preterm birth. Hypothesised mechanisms linking high ambient temperatures and heat waves (Variable X) to adverse maternal and neonatal outcomes, specifically preterm birth (Variable Y).

The primary hypothesis of the Bio-HEAT study is that exposure to high ambient temperatures during pregnancy is associated with elevated pro-inflammatory cytokine levels, such as interleukin-6 (IL-6) and other inflammatory markers, driven by heat-induced changes in gene expression, such as transcriptomic and epigenetic signatures. Figure 1 below demonstrates the plausible pathways we are investigating.

We have four secondary hypotheses:

- *Heat and systematic changes*: High ambient temperatures are hypothesised to disrupt endocrine function, enhance oxidative stress and inflammation, and increase the release of heat-shock proteins during pregnancy.
- *Interacting factors:* We hypothesise that multiple interacting factors, such as oxidative stress, altered cytokine profiles and psycho-social stressors contribute to the risk of preterm birth. Our analysis will account for these variables, assessing how they may mediate the relationship between heat exposure, and adverse maternal and neonatal outcomes. Furthermore, as psychosocial factors may evolve over pregnancy, we hypothesize that these fluctuations could influence health outcomes, such as preterm birth, and that these links may be accentuated by heat exposure.
- *Pathophysiological mechanisms in labour:* Heat exposure during labour is hypothesised to increase labour duration due to dehydration, and interventions such as hydration and cooling could reduce these effects.
- *Breastfeeding and heat exposure:* We hypothesise that heat exposure will reduce breastfeeding duration and frequency, and increase the sodium content of breastmilk primarily due to dehydration and discomfort experienced by both mother and infant during breastfeeding.

### Global health relevance of this study

Understanding the biological mechanisms and social vulnerabilities underpinning heat-health associations in pregnancy is critical in the context of rising global temperatures and the need for adaptive interventions. Adverse pregnancy outcomes, such as preterm birth, significantly influence health and developmental trajectories throughout the life course. By elucidating the links between heat exposure, inflammatory pathways, and preterm birth—and identifying actionable factors to mitigate these risks—this research has the potential to yield substantial public health benefits.

**Figure 2.**
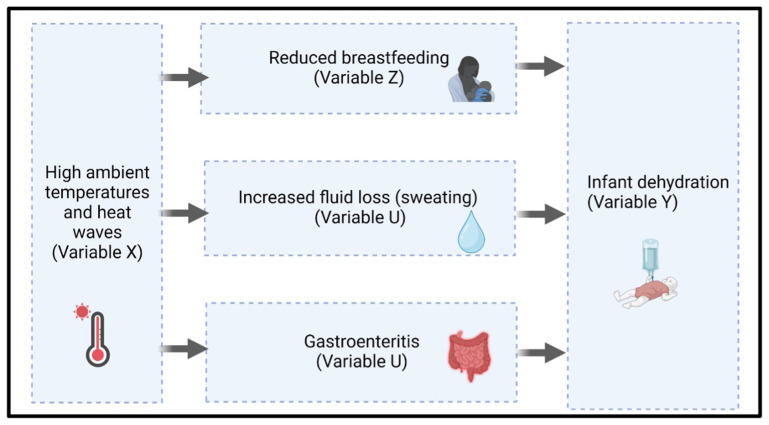
Proposed biological pathways from heat exposure to infant dehydration. Hypothesised mechanisms through which high ambient temperatures and heat waves (Variable X) may contribute to infant dehydration (Variable Y).

Additionally, examining how heat exposure affects hydration status and adverse outcomes during labour, and evaluating interventions to address these concerns, is essential. Breastfeeding, a cornerstone of child health, also warrants investigation to determine how climate-induced heat exposure may impact breastfeeding patterns and breastmilk composition. Figure 2 depicts three mechanistic pathways that we hypothesise contribute to the biological vulnerability of infants to dehydration.

Bio-HEAT will deliver detailed research findings on the relationships between heat exposure and biological vulnerabilities, with a focus on preterm birth, heat stress during labour, and breastfeeding. These insights will inform the development of conceptual frameworks that integrate the perspectives of affected women, ultimately guiding interventional research to address key modifiable factors in these pathways.

## Methods

### Study setting

The study will be conducted at a public regional hospital in Johannesburg which offers specialised maternal and paediatric care. The study site is situated in a high-density urban environment characterised by an Urban Heat Island effect, where temperatures can be significantly higher than the surrounding areas. Most residents in this area lack access to air conditioning, active or passive cooling systems, and urban greenery, exacerbating their heat vulnerability. Additionally, indoor cooking practices that involve combustion of solid fuels may augment heat exposure and degrade indoor air quality, complicating the health risks associated with high temperatures. Against this backdrop, Bio-HEAT is well-positioned to provide valuable insights into the causal pathways underpinning heat-health impacts in pregnant women and infants that may be generalisable to similar urban environments globally.

### Study design and participants

Bio-HEAT is a prospective cohort study designed to collect comprehensive data on heat exposure and its biological impacts on pregnant women and infants. Participants will be recruited from the regional hospital’s antenatal clinic, targeting women attending their 12-week nuchal translucency scan. Additional participants may also be recruited from the gynaecology ward, and other antenatal clinics in the catchment area as needed.

Participants will be followed from week 12–16 of pregnancy through to 12 months postpartum. The study comprises an overarching cohort and three nested sub-cohorts, purposively recruited from the overall cohort, each addressing specific research questions. Sample sizes as well as inclusion and exclusion criteria, are detailed below.

*Overall cohort:* Biological impacts of heat exposure on pregnant and postpartum women and their infants (N=200). Inclusion criteria for the overall cohort are as follows:

•   Gestational age of 12–16 weeks at enrolment based on ultrasound assessment of gestation

•   Aged 16 years or older

•   Resident in the hospital catchment area (hospital surrounds)

•   Intending to give birth at the study site and remain in the catchment area for the duration of the study

•   Able and willing to give informed consent

•   Agrees not to enrol in another study that may interfere with the results and interpretation of findings in this study

Exclusion criteria include:

•   Critical illness at enrolment, defined as hospital admission or at the discretion of the research doctor

•   Planning to deliver at an alternative maternity facility

•   Multiple pregnancy

•   Pregnancy with suspected or confirmed congenital anomaly prior to enrolment

*Sub-cohort 1:* Enhanced individualised activity and heat exposure among the highest-risk women (N=50) will include women with two or more of the following risk factors:

•   High heat exposure: Resides in low-cost housing, or has occupational heat exposure (e.g., working in kitchens or outdoor labour)

•   Any two of the following:

○ Pre-existing maternal health condition, including chronic hypertension, diabetes, renal dysfunction, autoimmune diseases, thyroid dysfunction, obesity (BMI>=30) or HIV infection
○ Maternal age–teenage pregnancy (age<20) or primiparous at ≥35 years old

*Sub-cohort 2*: Psychosocial factors along the pathway from heat exposure to prematurity (N=20–25). Serial qualitative interviews involving two groups of women, with final sample determined by data saturation:

•   Antepartum group: 10–15 women between 12–16 weeks gestation

•   Postpartum group: Up to 10 women who have had a preterm birth

*Sub-cohort 3*: Heat exposure and breastfeeding (N=50).

•   Participants will be conveniently sampled from the overall cohort with no additional selection criteria applied.

### Sample size considerations

Biological pathways analyses involve consideration of a wide range of outcomes. We calculated sample size to test the primary hypothesis using binary outcomes as a conservative approach. Continuous outcomes will offer considerably greater statistical power.

The primary inflammatory outcome interleukin-6 (>1000pg/ml) is strongly linked with preterm birth. With 200 women, two measures per participant, and an assumed 15% specimen loss, the study has >80% power to detect an 8% difference in the primary outcome. This calculation assumes that 5% of women exposed to the lowest quartile of maximum temperatures at a 0–2 day lag will exhibit raised interleukin-6 levels.

### Heat exposure monitoring

Monitoring heat exposure involves three components:

•   Facility Monitoring: The study site will be equipped with indoor temperature sensors (HOBOnet wireless sensor) to monitor the environments where the participants receive care, including the antenatal clinic and labour ward.

•   Community Monitoring: An outdoor weather station (HOBO MicroRX), covering the entire catchment area, will be installed on the premises of the facility to accurately capture temperature exposure in the area.

•   Personal Monitoring: Participants will wear wrist-mounted thermometers (ibuttons) throughout the study to unobtrusively and continuously record temperature and humidity exposure, delivering real-time, individual-level environmental data. To supplement this, geolocation of home addresses and pinned locations will be used to confirm housing types. Geolocated addresses will be securely linked with our existing geospatial grid data in Johannesburg, including economic status, vegetation indexes, temperature, air pollution, noise, ozone and other exposure variables.

### Study schedule

**Figure 3.**
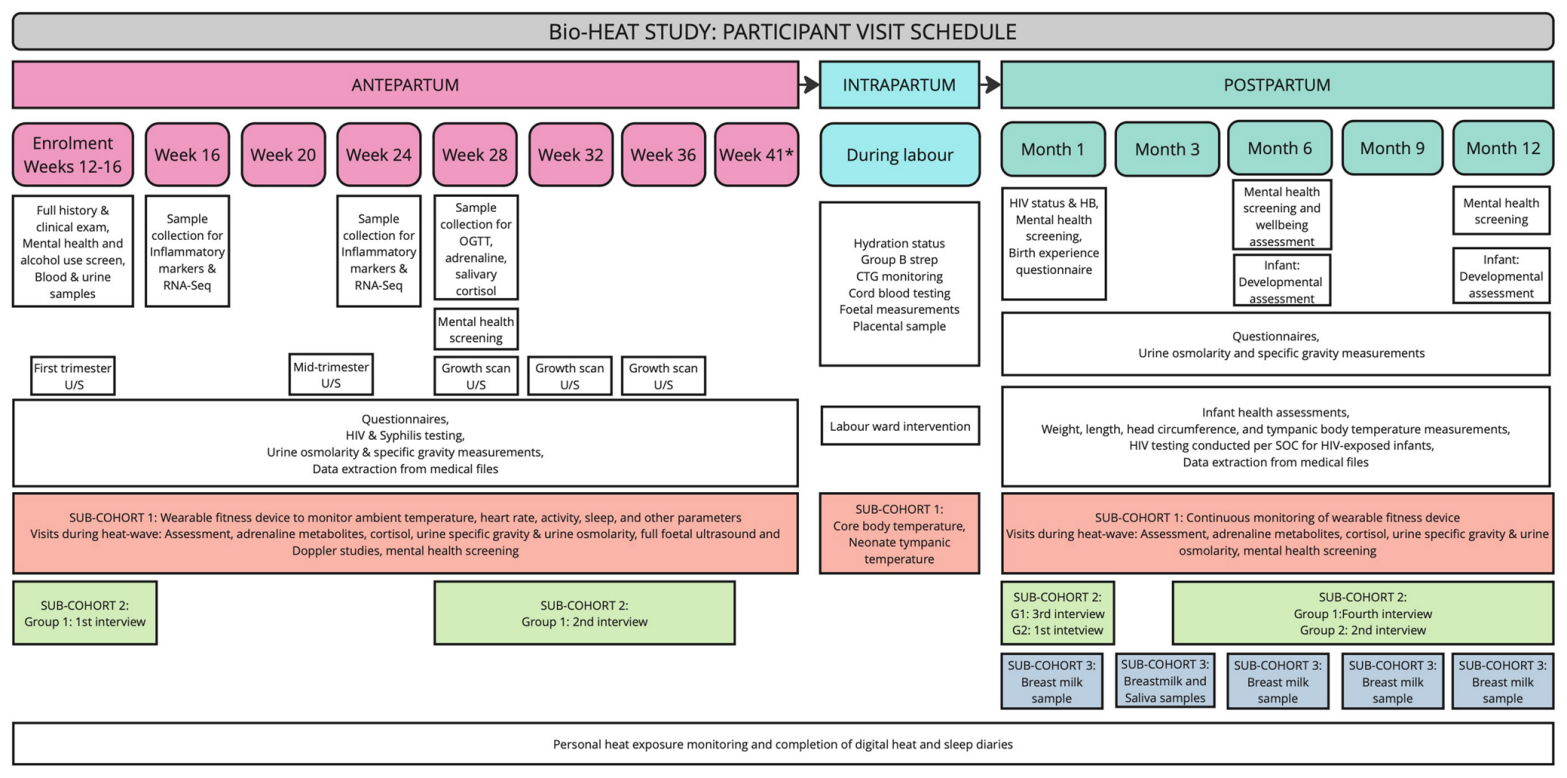
Summary of Participant Visit Schedule. Schedule of visits showing key assessments and unique sample collection across the antepartum, intrapartum, and postpartum periods for the overall cohort and the three sub-cohorts (Overall cohort- white; Sub-cohort 1- orange; Sub-cohort 2- green; Sub-cohort 3- blue).

The study schedule, summarised in Figure 3, includes a screening and enrolment visit, followed by antepartum follow-up visits every four weeks during pregnancy (at 16, 20, 24, 28, 32, 36, and 41 weeks- for those still pregnant) and an intrapartum visit. Postpartum follow-up visits will occur at 1, 3, 6, 9, and 12 months. Whenever possible, study visits will be coordinated with routine antenatal care appointments or baby wellness checks. The research unit is conveniently located near the hospital labour and gynaecology wards, ensuring easy access for participants. A window of +/- 14 days will apply to all scheduled visits, except during heat waves, defined as a maximum temperature >95th percentile for 2 or more consecutive days, when an additional study visit will be scheduled for selected participants within a 2-day window of the heatwave. Participants will be provided with a contact number for the study staff for questions, complaints or to inform the study staff of labour or any complications or adverse events.

### Enrolment visit

***Overall cohort.*** Following enrolment, participants will be asked questions on a range of sociodemographic factors, medical and obstetric history, behaviours related to heat adaptation and hydration (e.g., access to water and cool spaces, hydration practices, heat-related behaviours), mental health conditions and habits (substance use and activity levels). We will use validated tools including an Adapted Timeline Alcohol Use Calendar and the Alcohol Use Disorders Identification Test (AUDIT) to assess alcohol use ^31^, the Whooley antenatal depression and anxiety screening tool, the Edinburgh Postnatal Depression Scale (EPDS), the Primary Care Post Traumatic Stress Disorder (PTSD) screening tool to assess mental wellbeing, and the Pittsburgh Sleep Quality index to assess sleep. Each of these tools are available for use without further permissions required. Other relevant data will be extracted from the maternity case record to reduce participant burden.

The participant will be examined clinically, including blood pressure measurements, weight and height, and a general and system-specific examination, followed by a foetal ultrasound. If not already conducted at the antenatal clinic, blood will be drawn to test for renal function, thyroid function, HBA1c, a full blood count, and infections (HIV and syphilis). Cotinine testing will be done to assess nicotine exposure. A mid-stream urine sample will be collected to test for markers of dehydration, specifically osmolarity and urine-specific gravity.

Women will be given a personal wearable thermometer (ibuttons mounted on silicone wrist bands) and will be encouraged to regularly complete a WhatsApp based digital heat and sleep diary on their phones.

***Sub-cohort 1: High-risk women with individualised activity and heat exposure monitoring.*** The participants included in this sub-study will be additionally equipped with a wearable fitness device (Garmin Vivosmart 5) to facilitate continuous measurement of heart rate, activity, sleep, and other metrics.

***Sub-cohort 2: Psychosocial factors.*** Women in this sub-cohort will complete their first in-depth interview at a convenient time after their enrolment visit and before the 2 ^nd^ study visit. The interviews will be structured around three key areas: 1) socio-economic circumstances, 2) psychological circumstances, and 3) experience of weather and heat. These aspects will be explored initially and reassessed at subsequent interviews, with a focus on changes over time, weather, and the post-birth period. The interview guide is designed to be flexible, enabling questions to be repeated or further explored during each interview. Interviews will be audio-recorded and transcribed continuously for ongoing analysis. The research team will meet weekly to review transcripts and ensure data saturation is reached. Each participant will have a file containing written notes and transcripts for the team to review in advance of each interview.

### Antepartum study visits

***Overall cohort.*** As described above, antepartum study visits will take place 4-weekly between 16 and 41 weeks. At each visit we will repeat the questionnaires on heat adaptation and hydration as well as the sleep quality index. Mental health screening will also be conducted at visit 5 through the use of the following publicly available tools: The Patient Health Questionnaire (PHQ-9), the General Anxiety Disorder questionnaire (GAD-7), the World Health Organisation Disability Assessment Schedule (WHODAS-12). Each of these tools assess different aspects of mental wellbeing, complementing the other tools administered at enrolment and offer a multidimensional and holistic assessment of mental wellbeing. Study staff will review the data from wearables, and sleep diaries, assessing completeness, accuracy and quality.

Blood samples will be taken to test for HIV and syphilis if not already done at the antenatal clinic. At 16-and 28 weeks’ gestation, additional blood samples will be collected for inflammatory markers and RNA-Seq gene expression and stored for future analysis. Between 24–28 weeks, an oral glucose tolerance test will be performed, and samples collected to assess adrenaline metabolites (metanephrine and Vanillylmandelic Acid (VMA)). Participants will also provide an early morning saliva sample for cortisol and a midstream urine sample to assess dehydration markers (osmolarity and specific gravity). If dehydration is indicated, participants will be advised to increase fluid intake. A mid-trimester ultrasound will be conducted between weeks 20–24 and follow up growth scans at 28, 32, and 36 weeks. Additional scans may be conducted if clinically indicated.

***Sub-cohort 2: Psychosocial factors.*** Women in this sub-cohort will be interviewed again in their last trimester to provide insights into the changing nature of psychosocial circumstances and relationships, as well as how climate may have influenced these.

### Intrapartum study visit

***Overall cohort.*** During labour, the participant will be encouraged to continue wearing their personal wearable temperature monitoring device. Study staff will document the participant’s hydration practices and measure hydration status through urine osmolarity, urine specific gravity and blood tests (urea, creatinine and electrolytes). In addition, a vaginal and rectal swab to test for group B streptococcus colonisation will be conducted during labour. Lastly, the women and their foetus will be monitored continuously using a cardiotocograph as part of the routine standard of care and within the confines of the available resources within the labour ward at the hospital. Labour progress, mode of delivery, maternal and foetal morbidity, Apgar scores, foetal weight, length, and head circumference, and additional relevant information from the maternity case records and cardiotocograph will be documented.

Following labour, placentas will be collected according to site-specific standard operating procedures (SOPs). Placentas will be sent for histological analysis using the Amsterdam classification system which defines four major patterns of placental injury, namely: maternal vascular malperfusion, foetal vascular malperfusion, acute chorioamnionitis, and villitis of unknown aetiology. Placental membranes will also be sent for microscopy, culture and sensitivity analysis (MC&S). Where feasible, a sample of cord blood will be collected to test for biochemical markers of foetal stress.

***Sub-cohort 1: High-risk women with individualised activity and heat exposure monitoring.*** During labour, this sub-cohort will also wear core body thermometers attached to the chest, if clinical care permits. Alternatively, repeated core body temperature measurements will be recorded within the standard of care during labour, for instance, during theatre procedures where all electronic devices must be removed. The neonate will have a core body temperature surrogate measurement (e.g. tympanic temperature) taken immediately post-delivery, and at 15-minute intervals thereafter for an hour after delivery. This will be used to inform the likely intrauterine temperature, due to limitations in measuring intrauterine temperature.

### Labour ward intervention

We will collaborate with health workers to explore potential cooling and hydration interventions, guided by data from our ongoing and previous adaptation studies ^16^. Intervention selection will involve a literature review and 2–3 consultative meetings between health workers in the maternal child health units, external experts and the study team. Interventions may include increased access to cool water from water coolers installed in the ward, evaporative cooling technologies, such as fans with damp cloths, and installation of shading structures on the windows in the labour ward. Once the interventions have been selected, we will design an intervention evaluation detailing the interventions and the evaluation methodology. As part of the intervention evaluation, we plan to compare labour duration, women’s hydration levels and other relevant outcomes in women who received the interventions against historical data of labour outcomes in the facility prior to the study commencement.

### Postpartum study visits

***Overall cohort.*** During these visits, which will take place from month 1 and every 3 months post-delivery, the following questionnaires will be repeated: heat adaptation and hydration (every visit), substance use and activity levels (visits 1, 3, 5), and the sleep quality index. A questionnaire on breastfeeding practices will also be completed at every visit. Study staff will collect measurements (e.g., height, weight, waist, hip, and mid-upper arm circumference) and vital signs. At month 1, socio-economic conditions, mental health, and medical history will be reassessed and a birth experience questionnaire will be administered. Blood will be collected to check the participants’ haemoglobin and HIV status. Mental health screening will be repeated at months 6 and 12. Monitoring of digital heat and sleep diaries will continue throughout. Urine samples will be collected at every visit to check hydration levels.

Infant follow up: History of any infant episodes of gastroenteritis or hospital admissions, as well as milestone achievements will be obtained from the mother. Data on vaccinations, admissions and any other relevant information will be extracted from the *Road to Health Card* (child health record) during each visit. Weight, length and head circumference will be measured at each visit and body temperature measured using a non-invasive tympanic temperature monitoring device. At month 6, the Ages and Stages neurodevelopmental screening questionnaire will be completed. In addition, at month 12 a developmental assessment will be conducted for all children using the Bayley Scales of Infant and Toddler Development (BSID). HIV testing will be conducted as per the standard of care for HIV-exposed infants, and results extracted from the maternity case record. In the case of a foetal/neonatal or infant death, the postpartum women will be offered to continue her follow-up and referred for counselling, if required.

***Sub-cohort 1: High-risk women with individualised activity and heat exposure monitoring.*** Monitoring of the wearable activity device will continue throughout the postpartum period for these participants.

***Sub-cohort 2: Psychosocial factors.*** Women will be interviewed shortly after delivery and later during the first year of the infant’s life. Women can also engage with the study team at their other visits to provide further details about their experiences or if any unforeseen events occur such as admissions, premature delivery or other unexpected pregnancy outcomes, if they wish to.

***Sub-cohort 3: Breastfeeding.*** The dose-to-mother deuterium dilution method described below will be used in this sub-cohort at month 3 postpartum to assess breastmilk volumes and changes over time. Deuterium is a stable isotope of hydrogen that does not emit potentially harmful radiation ^32^. Deuterium and other stable isotopes have been used extensively for over half a century in metabolic studies to assess body composition, energy expenditure and protein turnover ^32, 33^. The deuterium that will be consumed by mothers in this study will enrich body water to a maximum of 0.1% in the mother and less than half of this in her infant ^34^. This is the same dose that has been used previously in several studies conducted in at least five African countries, which include Benin, Central African Republic, Morocco, Tanzania and South Africa. No adverse side effects have been reported at these low dose levels, and this method is not considered to be harmful for participating women or their infants.

The dose-to-mother deuterium dilution method involves the participant drinking a 30g dose of deuterium oxide, which mixes rapidly with her body water ^35^. The participant will be asked to rotate a small ball of cotton wool around their mouth until it is completely soaked with saliva. The cotton ball will then be placed in a 10-mL sterile disposable syringe and the plunger depressed to transfer the saliva into a 3.6-mL sterile cryovial. Saliva samples from the infant will be collected by a trained technician using a cotton wool swab enhanced with additional cotton wool. The collection process involves allowing the infant to suck on a dummy fitted with a cotton ball until it becomes fully saturated with saliva. Saliva samples will be taken from the mother and infant prior to the dose and self-collected by the mother over a 14 day period thereafter (day 1, 2, 3, 4, 13 and 14) ^35^. Women will be given pre-labelled cryovials with their unique participant identification number and dates. Women will be asked to record the time of sample collection on the cryovial.

At each visit, approximately 10 mL of breast milk will be collected from both breasts by maternal manual expression ^36^. Samples will be collected into a sterile container and stored in a temperature-controlled environment (between 4 to -20℃) until analysis. Sodium and potassium concentrations will be measured in whole breast milk by flame photometry on a digital flame photometer (IL 943, Analytical Instruments, LLC, USA) using Filteau’s or similar method. Briefly, breast milk will be centrifuged to separate the aqueous fraction, which will then be analysed for sodium and potassium levels. Equipment will be calibrated using standard solutions of known concentrations to ensure accuracy. All procedures will adhere to standardised protocols to minimise variability between measurements.

### Additional study visits during heatwaves

***Sub-cohort 1: High-risk women with individualised activity and heat exposure monitoring.*** Visits during heatwaves will involve assessment of heat adaptation, access to water and cool spaces, hydration practices, heat-related behaviours, mental health screening and sleep quality. Physical examination will include height, weight, mid-upper arm circumference (MUAC) and foetal ultrasound. Bloods will be drawn for inflammatory markers, RNA gene sequencing, renal function, adrenaline and cortisol and urine collected for urine osmolarity, urine specific gravity. Temperature and activity data from wearable devices will also be reviewed. 

***Sub-cohort 3: Breastfeeding.*** Breast milk will be collected as previously described.

### Data analysis

***Overall cohort.*** Analysis will account for clustering from repeated measures where relevant, and adjust for seasonal and sub-season patterns to reduce biases from acclimatisation or acclimation.

Individualised temperature and humidity exposures for women will be modelled from continuous measures of personal temperature exposures as well as more complex climate measures such as wind speed and radiant temperature from household, maternity facility and community weather stations, allowing calculation of cumulative heat stress exposure as well as identification of points of extreme heat stress exposure ^37, 38^. Heat stress exposure will be calculated using: Wet Bulb Globe Temperature (WBGT), Universal Thermal Climate Index (UTCI), and Apparent temperature (AT) which have been used extensively in Southern Africa ^21, 39, 40^, as are appropriate for high incoming solar radiation, higher temperatures and average relative humidity conditions.

Biomarker analysis will be conducted using multivariate regression models to examine the association between heat exposure and inflammatory markers, adjusting for potential confounders. Inferential analysis of longitudinal data will include mixed-effects linear models. Our omics approach, to enhance precision, includes assessing maternal transcriptomic and epigenetic signatures, using serial RNA-Seq data, to track up-and down-regulated genes before-and- after heat extremes. The read-count matrix for differential gene expression and pathway analyses will be produced through the nf-rnaSeqCount pipeline ^41^, using the raw RNA-Seq data from the different time points. Similarities and differences between RNA-Seq samples will be assessed through Principal Component Analysis (PCA). Different tools, including DESeq2 and edgeR packages in R, will be used to identify significant gene expression changes related to heat exposure. To visualise the significantly differentiated genes between the different time points, heatmaps and volcano plots will be created. Pathway analyses of the significantly expressed genes will be carried out using gage and pathview packages in R. Analyses will be presented overall, and by prespecified population sub-groups (to identify groups for future targeted interventions). Bonferroni corrections will be applied to counteract potential multiple testing biases.

***Sub-cohort 1: High-risk women with individualised activity and heat exposure monitoring.*** During the analysis, two components related to heat exposure will be explored. Firstly, differential heat stress experienced in different environments, including the community, facility and other areas captured by the personal thermometers will be compared and contrasted using summary statistics as described in the overall cohort above. Wearable activity data will be post-processed to account for no-wear time, including thresholds on inactivity and discarding steps not associated with heart rates. Linear mixed effects models will be used to assess the impact of heat exposure on 1) activity (number of steps and % sedentary time) and 2) sleep (duration and quality) ^42, 43^. Second, overall heat stress and heat strain experienced by the individual will be modelled based on the integration of multiple data sources, including the various environmental and physiological variables (e.g., using step counts and heart rate as surrogates), as well as the physiological vital signs such as core body temperature and heart rate of the individual. These inputs are vital in assessing common heat strain indices such as the Physiological Strain Index (PSI), which has been utilised in similar studies on biological pathways of heat exposure ^44^, recorded by wearable devices and weather stations. This index can then be used as a reference in the statistical analysis of the biological end-points which are more detailed physiological responses to heat.

***Sub-cohort 2: Psychosocial factors.*** Data from in-depth interviews will initially be analysed separately, blinded to the quantitative data, using a non-positivist, phenomenological approach to explore the biopsychosocial experiences of pregnant women and construct pathways around pregnancy outcomes. Quantitative data (psychological and socioeconomic) will be analysed to identify associations and causal pathways. The qualitative and quantitative data will then be integrated, comparing interview insights with questionnaire responses to validate the tools and identify gaps or discrepancies. Similarly, discussions on heat experiences will be compared to quantitative thermal recordings to ensure robustness. Key aspects, including mental health, social circumstances, food insecurity, relationships, biological events, and hospital interactions, will be cross-checked. A grounded theory approach will be used with an emphasis on avoiding assumptions about suspected causal pathways. Analysis will take the form of coding the text and using a constant comparative method of analysis.

***Sub-cohort 3: Breastfeeding.*** Deuterium enrichment levels in the saliva of both the mother and the infant will be analysed relative to their pre-dose samples using a Fourier Transform Infrared Spectrometer (FTIR). Model curves will be fitted to these values using the open-source statistical software R to estimate the infant’s breastmilk intake and, by extension, the mother’s breastmilk output.

Box and whisker plots will be used to give a graphical comparison of the breast milk volume, for all women, but then also by different categories of temperature (e.g. quartiles). Multi-level regression analyses will be conducted to explore the relationship between breast milk volume, and outdoor, household and personal temperature. Breast milk sodium and potassium levels will be assessed for normality. If data are close to symmetric, they will be summarised by measures of central tendency. If they are skewed, they will be log transformed and summarised as geometric means. Multilevel regression models will be used to investigate whether the sodium content of breast milk is affected by ambient and personal temperatures, and to explore the relationship between self-reported breastfeeding and temperature. Analysis will account for potential confounding factors including maternal age and co-morbidities.

## Participant safety

### Research staff training

All staff will undergo comprehensive training before beginning work on the study, covering ethics, informed consent, protocol-specific procedures, and any qualifications required for their specific roles.

### Routine antenatal care and delivery

Study participants will be retained at the hospital site for routine antenatal care and delivery. Participants requiring emergency care or outpatient visits will be referred to the hospital. Continuous communication will be maintained between hospital and study staff throughout.

### Risks

Participation in the study involves minimal risks and study participants remain under the care of the research hospital site throughout. Potential risks and mitigating strategies include:

•   Psychological risks: Pregnancy and infant related studies have the potential for psychological stress, particularly if there has been an adverse outcome, e.g., preterm birth, miscarriage, stillbirth, or infant death. Research staff will be trained to recognise signs of psychological distress and will be instructed on how to assist participants in obtaining support. A distress protocol, aligned with the current practices in place, will be followed in these instances.

•   Risk of vulnerability to theft with wearables: In South Africa, wearing an activity tracker on the wrist may increase the risk of becoming a target for theft. Based on our experience with a similar study outside of Johannesburg, participants did not encounter this issue. However, we appreciate the different study settings in Johannesburg. We will counsel the pregnant women about the risks and communicate with our Community Advisory Board about mitigating the risk.

•   Physical risks: Study activities that involve any physical interventions or procedures (e.g., phlebotomy or ultrasound), will be performed by qualified personnel in a setting equipped to handle and respond to any adverse reactions. Regular monitoring will ensure that any signs of distress or adverse effects are quickly addressed.

## Quality assurance

High standards of quality will be achieved by full adherence to SOPs. Any deviations from the protocol or instances of non-compliance with ethical standards or unanticipated problems will be recorded. The PI will be immediately informed and appropriate corrective and preventive actions will be implemented. A corrective and preventive action log will be maintained to track these actions and their outcomes, and these will be reported to our ethics board and the Wellcome Trust as appropriate.

## Data management

We will follow International Council for Harmonisation-Good Clinical Practice (ICH-GCP) guidelines including using unique participant identification numbers, securely storing both hard copies and electronic versions of participant data and restricting access to trained research staff. The study will adhere to the Wellcome Trust regulations on data sharing and relevant local data protection laws, such as the South African Protection of Personal Information Act (POPIA). External access to the data collected in this study will be partly restricted – i.e., the dataset will be shared only upon written application to the study PIs. Data to be used for other research studies, including studies on a different topic, may be shared subject to ethics and PI approval. This applies to additional research or analyses not mentioned in the study protocol or with collaborators not part of the protocol team.

### Data collection

Data will be collected using a combination of electronic data capture systems and paper-based forms. This will include standardised questionnaires, automated logging of wearable data, WhatsApp-based diaries and digital recording devices. All equipment used in the study, including data collection and monitoring devices, will be regularly calibrated and maintained according to the manufacturer's specifications to ensure accurate and consistent data collection. Range checks and automated validation rules will be implemented at the point of data entry to minimise errors. Additionally, electronic data will be cross-checked against source documents to ensure accuracy.

### Data storage and backup

The electronic data will be stored in a secure, password-protected environment that adheres to international data protection standards, such as REDCap (Research Electronic Data Capture - https://project-redcap.org), Microsoft SharePoint, or Microsoft Azure. Large RNA-Seq data will be stored (and analysed) on the University of the Witwatersrand Compute Cluster. Electronic data will be backed up on both onsite and offsite encrypted servers. Physical forms will be securely stored in locked cabinets within a restricted-access area at the study site. Upon conclusion of the study, all records will be retained for at least ten years per institutional and funding body guidelines. Deidentified data will be archived in a secure, encrypted format in institutional repositories for long-term preservation. At the same time, physical records will be securely shredded through certified destruction services to protect sensitive participant information.

## Dissemination

We will ensure that the results are accessible to all stakeholders, including the scientific community, participants, healthcare providers, and policymakers. Findings will be published in open-access, peer-reviewed journals and presented at scientific conferences and expert meetings to advance academic discourse and professional education. Public communications, such as press releases issued through the University of the Witwatersrand and other media channels, will help raise awareness and understanding among the general public. Participants and local communities will receive simple, non-technical summaries of the findings, tailored to appropriate languages and formats, including leaflets, posters, and online content. These materials will be reviewed with the Community Advisory Board to ensure relevance and clarity before distribution. Key stakeholders, including community leaders, advocacy groups, and professional organisations, will be actively engaged to ensure the findings inform policy development, advocacy efforts, and intervention strategies at local, national, and international levels. Fully de-identified data will be made available upon publication of the final manuscript planned within Bio-HEAT. These datasets may be placed in repositories, and will be made available upon reasonable written request including confirmation of required ethical approval and completion of a Data Transfer Agreement. Code used for analysis will be shared with each manuscript as a supplementary file.

## Ethics and consent

The study adheres to ethical guidelines outlined in the Declaration of Helsinki ( https://www.wma.net/policies-post/wma-declaration-of-helsinki-ethical-principles-for-medical-research-involving-human-subjects/). Written informed consent will be obtained from all participants. Study staff will emphasise voluntary participation and assure participants that provision of routine antenatal care will not be affected. Participants will be encouraged to discuss their involvement with household members before making a decision. If a participant is unable to speak or understand one of the local languages available for the informed consent process, an impartial witness will be allowed to translate. Separate Informed Consent Forms (ICFs) will be provided for sample storage and genetic testing. For participants under 18, consent will be obtained from a parent or guardian, and the minor will complete an assent form. Participants will have the right to withdraw from the study at any time without any penalty. Further data will not be collected from a withdrawn participant, however data already collected will be retained.

### Ethics approval

The study protocol has received ethical approval from the University of the Witwatersrand Human Research Ethics Committee (ethics ref 240804, dated 9 ^th^ October 2024) as well as facility and district approval through the Hospital review committee (28 ^th^ January 2025) and the Research Committee of Johannesburg Health District (18 ^th^ November 2024). 

## Data Availability

No data are associated with this article.
