# Supplementary material for: Study protocol for the Bio-HEAT study: Investigating the Biological pathways from HEAT exposure to preterm birth and other adverse maternal and child health outcomes in South Africa
Source: Wellcome Open Res. 2025 Jun 6;10:121. Originally published 2025 Mar 5. [Version 2] doi: 10.12688/wellcomeopenres.23616.2 (PMC12209768; doi:10.12688/wellcomeopenres.23616.2)
Supplement: Peer review history

# Reviewer report: Reviewer response for version 2

**DOI:** 10.21956/wellcomeopenres.26873.r126576

**Published:** 2025-06-30

**Authors:** Santosh Pandipati

**Affiliations:** 1 Lōvu Health and Silicon Valley Maternal-Fetal Medicine, Los Gatos, California, USA; 2 Obstetrix of San Jose, e-Lōvu Health, e-Lōvu Health, USA

This is an incredibly exciting research study looking at uncovering mechanisms of excess heat exposure on pregnancy and postpartum outcomes. To my knowledge, such a comprehensive longitudinal study has not been performed. I am especially impressed at the evaluation of both serum markers as well as psychosocial determinants that may contribute to adverse outcomes.

 I believe the dataset to be collected will be vast, and I suspect multiple publications spiraling out of this study. I am especially impressed with the comprehensiveness of the data collection, ranging from facility to community to personal monitoring, and looking not just at physical, biochemical, and gene expression parameters, but also at the psychosocial aspects well into postpartum health, including newborn health (vis-à-vis breastfeeding). 

 It will be good to perform standardized mental health assessments more often throughout pregnancy and postpartum using PHQ-9 or EPDS surveys (not just at 5 months). Additionally, I would highly recommend administering such surveys during heatwave visits. This is not written out in the text, but it seems to be listed in Figure 3, and hence my request for clarification. There is a clear correlation between untreated mental health conditions such as depression and higher rates of preterm birth. There is also clear correlation between climate stressors such as heatwaves and deteriorated mental health. So, it will be interesting to see if there is an interplay between mental health, climate, and outcomes.

 Finally, as climate continues to change, it may be good to compare outcomes against a historical comparison if outcome data is available - i.e., compare this study population’s overall outcomes to the outcomes of historical controls from the year prior, 5 years prior, and 10 years prior. Of course, prior historical controls will not have all the data these researchers will be collecting in their study, but it will place some historical context for the study population. 

 Overall, I am excited to see what comes out of this study.

Is the study design appropriate for the research question?

Yes

Is the rationale for, and objectives of, the study clearly described?

Yes

Are sufficient details of the methods provided to allow replication by others?

Yes

Are the datasets clearly presented in a useable and accessible format?

Yes

Reviewer Expertise:

Maternal-fetal medicine, climate change impacts to reproductive health, digital health and telemedicine.

I confirm that I have read this submission and believe that I have an appropriate level of expertise to confirm that it is of an acceptable scientific standard.

# Reviewer report: Reviewer response for version 1

**DOI:** 10.21956/wellcomeopenres.26054.r121795

**Published:** 2025-04-24

**Authors:** Claudia Hanson

**Affiliations:** 1 Karolinska Institutet, Stockholm, Sweden

Thank you very much proving me with the opportunity to review this important protocol. The hypothesis is clearly stated and the sub-hypothesis as well. Understanding physiological mechanisms will be of major importance to develop the best interventions for mitigation.

 I am missing a bit a consideration of why which measurements. For example, why which markers in pregnancy, cord blood (which measurement).  

 Further, I am missing a consideration of pregnancy complications including pre- / eclampsia, vaginal infection other then group B-streptococcus. Also, will you include information of complications at birth?

 The protocol is not well referenced, for example, there is no reference to the Amsterdam classification for placenta pathology.

 The quality assurance part does not go into sufficient details. You are collecting a large variety of variable, and different quality assurance mechanisms and SOPs will need to be applied. A similar concern is for the data processes and management. Probably a table on data management for the very different types of data may have been used.

 I am also not clear about patient involvement. I am happy to hear about the involvement of the labour room staff in respect to cooling, but I am less clear about patient involvement in the design of the study, eg. patients may raise additional important factors which are not yet included, such as work and commuting.

Is the study design appropriate for the research question?

Yes

Is the rationale for, and objectives of, the study clearly described?

Yes

Are sufficient details of the methods provided to allow replication by others?

Partly

Are the datasets clearly presented in a useable and accessible format?

Partly

Reviewer Expertise:

Obstetrics and women's health, epidemiology

I confirm that I have read this submission and believe that I have an appropriate level of expertise to confirm that it is of an acceptable scientific standard, however I have significant reservations, as outlined above.

# Reviewer report: Reviewer response for version 1

**DOI:** 10.21956/wellcomeopenres.26054.r121797

**Published:** 2025-04-23

**Authors:** Ashish KC

**Affiliations:** 1 University of Gothenburg, Gothenburg, Sweden

Thank you for inviting me to review this interesting study protocol on biological pathway on heat exposure to preterm birth and other maternal and child health outcomes. This is a very valuable study to provide the epidemiological explanation on heat events to preterm birth

 This study aim to investigate the environmental exposure to high ambient temperature on preterm birth and the causal pathway to exposure.

 I have some comments on the hypothesis driven for the study

 - Non-optimal temperature either lower than 50th centile or higher than 50th centile has been associated to poor obstetric outcomes including preterm birth, still birth and neonatal mortality https://www.nature.com/articles/s41591-024-03245-7 and Ref:1 indicating that non-optimal ambient temperature exposure has a corelation between heat and preterm birth. It would be worth to provide a note to it.

 - Even though there is a cumulative effect of non-optimal temperature to preterm birth, the lag effect i.e harvesting effect might be seen in preterm birth, can the researchers discuss about it

 -In the abstract researchers mention that "maternal inflammation will be tested by analysis the epigenetic changes associated with inflammatory cytokines protein and gene expression". Based on the studies from Genome wide association study and Mendenlian law, any genetic variant is always constant since conception, so the expression of cytokine is based on the dysfunction of the cytokine receptor dysfunction. https://doi.org/10.3390/ijms252011332.

 further not all peripheral inflammation will cause the trigger preterm labour, until the the inflammation cross the blood brain barrier, the inflammatory chain of reaction is required.

 Further, why is the focus on IL-6, there are a range of pro-inflammatory and anti-inflammatory cytokines which can cause BBB inflammation Ref:2

 Sample size estimation- I still did not get the basis of the sample size based on IL-6, has there been a study to estimate IL-6 >1000pg/ml is linked with preterm birth.

 In hypotheses, the researchers mention that psycho-social stressor is a mediator of preterm birth, but does it not also link with the increase in pro-inflammatory or neuro-hormonal disturbance. Can it be that there is a reversal phenomenon that due to increased pro-inflammatory genetic expression and disturbance in neuro-hormonal gene expression, that the social stress can change the pyschological response and thus preterm birth.

 Thank you once again for inviting me to review the protocol

Is the study design appropriate for the research question?

Yes

Is the rationale for, and objectives of, the study clearly described?

Yes

Are sufficient details of the methods provided to allow replication by others?

Partly

Are the datasets clearly presented in a useable and accessible format?

Not applicable

Reviewer Expertise:

Heat event to perinatal health

I confirm that I have read this submission and believe that I have an appropriate level of expertise to confirm that it is of an acceptable scientific standard.

## References

[1] : Effect of non-optimal ambient temperature on preterm birth stratified by social positioning in Nepal: A space-time-stratified case-crossover study. *Environ Res* .2024;258: 10.1016/j.envres.2024.119501 119501 10.1016/j.envres.2024.119501 38942260

[2] : Characterization of type 1 and type 2 cytokine production profile in physiologic and pathologic human pregnancy. *Clin Exp Immunol* .1996;106(1) : 10.1046/j.1365-2249.1996.d01-809.x 127-33 10.1046/j.1365-2249.1996.d01-809.x 8870710 PMC2200555

# Response

**Published:** 2025-05-27

**Authors:** Ijeoma Solarin

**Affiliations:** Wits Planetary Health Research (Wits PHR), University of the Witwatersrand Johannesburg Faculty of Health Sciences, Johannesburg, Gauteng, South Africa

Apologies, I tried to submit responses in a table format but the layout did not work. I am therefore reposting so the comments and responses display properly.  Response to reviewer: **Ashish KC **University of Gothenburg, Gothenburg, Sweden 1.1. *Thank you for inviting me to review this interesting study protocol on biological pathway on heat exposure to preterm birth and other maternal and child health outcomes. This is a very valuable study to provide the epidemiological explanation on heat events to preterm birth. This study aim to investigate the environmental exposure to high ambient temperature on preterm birth and the causal pathway to exposure.* **Author response: **Thank you for these comments and careful review. *1.2. I have some comments on the hypothesis driven for the study*

*- Non-optimal temperature either lower than 50th centile or higher than 50th centile has been associated to poor obstetric outcomes including preterm birth, still birth and neonatal mortality https://www.nature.com/articles/s41591-024-03245-7 and Ref:1 indicating that non- optimal ambient temperature exposure has a corelation between heat and preterm birth. It would be worth to provide a note to it.* **Author response: **Thank you for this helpful comment and for sharing the relevant references. We agree that both high and low ambient temperatures have been associated with adverse obstetric outcomes, including preterm birth, stillbirth, and neonatal mortality. While the primary focus of our study is on high temperature exposures—given their growing relevance in the context of anthropogenic climate change, the particular relevance in our study setting in sub-Saharan Africa and the urgent need to understand underlying biological mechanisms—we recognise the importance of non-optimal low temperature exposure as well. While the references shared by the reviewer (Kc et al. (2024) and Hanson et al (2024)) underscore the importance of considering both cold and hot non-optimal temperatures, even these studies report that heat exposure tends to have a more consistent and pronounced association with adverse outcomes than cold exposure, especially in low-resources contexts. We have nevertheless updated the manuscript to acknowledge this broader evidence base and will consider it when interpreting our findings. Added text in section on Global health relevance: *While both high and low ambient temperatures have been associated with poor obstetric outcomes (Kc et al., 2024, Nyadanu et al., 2024), this study focuses on heat exposure, which is of particular concern in our African contex*t. *1.3 Even though there is a cumulative effect of non-optimal temperature to preterm birth, the lag effect i.e harvesting effect might be seen in preterm birth, can the researchers discuss about it* **Author response: **Thank you for this comment. We appreciate the suggestion to consider a potential lag or “harvesting” effect. As we understand it, the harvesting effect typically refers to temporal displacement of events that would have occurred soon regardless, and it is most often applied in the context of mortality, particularly among older or frail individuals. We are not certain that this concept directly translates to preterm birth, as a shift from term to preterm delivery due to heat exposure may represent a clinically meaningful outcome that could have been avoided rather than temporal displacement of an outcome that was likely to occur regardless. Nonetheless, we acknowledge that further discussion on lag structures and potential timing effects is warranted and will consider this in our interpretation of findings. *1.4 In the abstract researchers mention that "maternal inflammation will be tested by analysis the epigenetic changes associated with inflammatory cytokines protein and gene expression". Based on the studies from Genome wide association study and Mendenlian law, any genetic variant is always constant since conception, so the expression of cytokine is based on the dysfunction of the cytokine receptor dysfunction. https://doi.org/10.3390/ijms252011332.* **Author Response: **Our approaches to examining heat exposure and inflammation includes assessing maternal transcriptomic and epigenetic signatures, using serial RNA-Seq data, to track up-and down-regulated genes before-and- after heat extremes. These are not Mendelian changes, but the differential gene expression and pathway analyses assessed using RNA Seq data. We can visualise the significantly differentiated genes between the different time points and see which genes are being expressed. We don’t do GWAS or similar genetic testing in this study, as the reviewer points out. 1.5 *further not all peripheral inflammation will cause the trigger preterm labour, until the inflammation cross the bloodbrain barrier, the inflammatory chain of reaction is required. Further, why is the focus on IL-6, there are a range of pro-inflammatory and anti-inflammatory cytokines which can cause BBB inflammation Ref:2* **Author reponse: **We appreciate your important point that not all peripheral inflammation will trigger PTB, and that specific inflammatory pathways, including those involving the blood-brain barrier, may play a role. However, our study is primarily focused on inflammatory pathways at the maternal-foetal interface (such as placenta, myometrium), which are more directly implicated in the initiation of labour than the BBB. We also acknowledge that IL-6 is a non-specific marker and that multiple cytokines, both pro- and anti-inflammatory, may contribute to the broader inflammatory response. Our focus on IL-6 is based on several considerations: 1). Evidence supporting its role in PTB; 2). its relative ease of measurement and low cost of testing; and 3). its widespread use in research, which facilitates comparison across studies. Nevertheless, we do plan on conducting a more comprehensive biomarker analysis using a panel of 96 inflammatory markers. This will allow us to investigate a range of cytokines implicated in both peripheral and central inflammation and inform more complex analysis. Biomarker analysis will be conducted using multivariate regression models to examine the association between heat exposure and inflammatory markers, adjusting for potential confounders. Inferential analysis of longitudinal data will include mixed-effects linear models. Much of this analysis is done using visualisations such as heatmaps and volcano plots. Bonferroni corrections will be applied to counteract potential multiple testing biases. We recognise that much remains unknown about the precise pathways - whether systemic, BBB, or at the level of the cervix and membranes - and which pathways are most important. This study will try address some of those questions. We have added reference to a recent review on heat and preterm birth Mushimiyimana 2025, which discusses this in more detail. 2.6 *Sample size estimation- I still did not get the basis of the sample size based on IL-6, has there been a study to estimate IL-6 >1000pg/ml is linked with preterm birth.* **Author response:** Many thanks for this query, the point made here is valid. We should have noted the rationale for selecting Il-6 as the outcome of interest. IL-6 is one of the most widely studied inflammatory markers and indeed, several studies have linked Il-6 with preterm birth, as in the papers we now cite for this: ( Prairie et al. 2021, Hatzidaki et al., 2005, Karampitsakos et al., 2025). In addition, IL-6 has been shown to be raised by heat ( Maloney & Duffy, 2024). We do however acknowledge that the originally specified IL-6 threshold of >1000 pg/mL was not appropriate for maternal serum. This was an oversight, and we appreciate the opportunity to correct it. We have revised this threshold in the manuscript to reflect more clinically and biologically relevant values for maternal serum which are much lower depending on presence of infection or specific population group being studied. While there is no universally accepted threshold for IL-6 in predicting PTB, and some variability exists across studies, we have revised our threshold to 10pg/ml as a conservative measure based on other studies which identify maternal serum IL-6 levels around this value as predictive of preterm birth (e.g., Murtha et al., 1998) and which was our original intent. We also acknowledge that we have made some assumptions due to the scarcity of existing environmental exposure literature in this area. Nevertheless, our assumptions are based on conservative and pragmatic estimates to ensure sufficient power. One of our aims is to contribute new evidence on the potential relationship between environmental temperature and maternal systemic inflammation. We have clarified this in the revised manuscript. Updated text in sample size calculation: *The primary inflammatory outcome interleukin-6 is strongly linked with PTB (Prairie et al., 2021, Hatzidaki et al., 2005, Karampitsakos et al., 2025). However, there is currently no universally accepted threshold for IL-6 in predicting PTB, with high levels of variability across studies. We have based our sample size on IL>10pg/ml as a conservative measure in keeping with other studies which identify maternal serum levels around this value as predictive of PTB (Murtha et al., 1998).* 2.7 *In hypotheses, the researchers mention that psycho-social stressor is a mediator of preterm birth, but does it not also link with the increase in pro-inflammatory or neuro-hormonal disturbance. Can it be that there is a reversal phenomenon that due to increased pro-inflammatory genetic expression and disturbance in neuro-hormonal gene expression, that the social stress can change the pyschological response and thus preterm birth.* **Author response:** Thank you for this thoughtful comment. We agree that psychosocial stress may not only act as a mediator but may also be shaped by underlying biological processes, such as pro-inflammatory and neuro-hormonal disturbances. We acknowledge the possibility of bidirectional, feedback loops or reverse relationships, whereby heightened inflammatory or neuroendocrine activity—potentially influenced by genetic expression—could amplify psychological vulnerability or stress perception. In our conceptual model, we view psychosocial stress, inflammatory pathways, and neuro-hormonal disturbances as potentially interacting and reinforcing components. While our study is not designed to fully disentangle these causal directions, our analyses will explore how these variables may individually and collectively mediate the association between heat exposure and preterm birth. We will also consider how these relationships may evolve over the course of pregnancy and be modulated by environmental stressors such as heat. 2.8 *Thank you once again for inviting me to review the protocol* **Author response:** Thank you for your valuable feedback.
